# Supplementary material for: Direct reciprocity between individuals that use different strategy spaces
Source: PLoS Comput Biol. 2022 Jun 14;18(6):e1010149. doi: 10.1371/journal.pcbi.1010149 (PMC9197081; doi:10.1371/journal.pcbi.1010149)
Supplement: S1 Data — Table A. Pairwise competitions under very weak selection. The table shows the same type of data as Table 1, but using a selection strength of β = 1 instead of β = 100. We find that lower memory always wins in a pairwise competition, regardless of the value of c. Simulations are run for T = 109 time steps. Table B. Wins and scores under very weak selection. The table shows the same type of data as Table 2, but again using a selection strength of β = 1 instead of β = 100. With respect to both measures, wins and scores, U is always ranked first. Simulations are run for T = 109 time steps. Table C. Self scores and combined scores under very weak selection. The table shows the same type of data as Table 3, but for β = 1 instead of β = 100. The space of memory-1 strategies tends to have the largest self-payoff, but unconditional strategies have the largest combined score across all cost values. Simulations are run for T = 109 time steps. Table D. Pairwise competitions under rather weak selection. The table shows the same type of data as Table 1, but using a selection strength of β = 10. Similar to the case of very weak selection, we find that lower memory always wins in a pairwise competition. Simulations are run for T = 109 time steps. Table E. Wins and scores under rather weak selection. The table shows the same type of data as Table 2, but using a selection strength of β = 10. Similar to the case of very weak selection, U is always ranked first. Simulations are run for T = 109 time steps. Table F. Self scores and combined scores under rather weak selection. The table shows the same type of data as Table 3, but with β = 10. Again, the space of memory-1 strategies tends to have the largest self-payoff, but reactive and unconditional strategies have the largest combined score. Simulations are run for T = 109 time steps. Table G. Pairwise competitions under very strong selection. The table shows the same type of data as Table 1, but using a selection strength of β = 1000. [file pcbi.1010149.s011.pdf]

Supplementary Data

Tournament: $\beta = 1$

Cost c	$\mathcal{M} : \mathcal{R}$		$\mathcal{M} : \mathcal{U}$		$\mathcal{R} : \mathcal{U}$		$(b - c)$
	$\bar{\pi}_{\mathcal{M},\mathcal{R}}$	$\bar{\pi}_{\mathcal{R},\mathcal{M}}$	$\bar{\pi}_{\mathcal{M},\mathcal{U}}$	$\bar{\pi}_{\mathcal{U},\mathcal{M}}$	$\bar{\pi}_{\mathcal{R},\mathcal{U}}$	$\bar{\pi}_{\mathcal{U},\mathcal{R}}$	
0.1	0.448	**0.450**	0.442	**0.452**	0.442	**0.453**	0.9
0.2	0.394	**0.398**	0.384	**0.402**	0.384	**0.400**	0.8
0.3	0.341	**0.347**	0.327	**0.353**	0.328	**0.350**	0.7
0.4	0.288	**0.297**	0.270	**0.305**	0.272	**0.300**	0.6
0.5	0.236	**0.248**	0.215	**0.259**	0.218	**0.253**	0.5
0.6	0.184	**0.200**	0.159	**0.215**	0.165	**0.207**	0.4
0.7	0.133	**0.153**	0.105	**0.173**	0.113	**0.162**	0.3
0.8	0.083	**0.106**	0.052	**0.131**	0.062	**0.119**	0.2
0.9	0.033	**0.061**	-0.001	**0.092**	0.012	**0.077**	0.1

Table A: Pairwise competitions under very weak selection. The table shows the same type of data as **Tab. 1**, but using a selection strength of $\beta = 1$ instead of $\beta = 100$. We find that lower memory always wins in a pairwise competition, regardless of the value of c. Simulations are run for $T = 10^9$ time steps.

Cost c	Wins				Score			
	$\mathcal{M}$	$\mathcal{R}$	$\mathcal{U}$	Ranking	$\mathcal{M}$	$\mathcal{R}$	$\mathcal{U}$	Ranking
0.1	0	1	**2**	$\mathcal{U}, \mathcal{R}, \mathcal{M}$	0.890	0.892	**0.905**	$\mathcal{U}, \mathcal{R}, \mathcal{M}$
0.2	0	1	**2**	$\mathcal{U}, \mathcal{R}, \mathcal{M}$	0.778	0.783	**0.802**	$\mathcal{U}, \mathcal{R}, \mathcal{M}$
0.3	0	1	**2**	$\mathcal{U}, \mathcal{R}, \mathcal{M}$	0.668	0.675	**0.702**	$\mathcal{U}, \mathcal{R}, \mathcal{M}$
0.4	0	1	**2**	$\mathcal{U}, \mathcal{R}, \mathcal{M}$	0.558	0.570	**0.606**	$\mathcal{U}, \mathcal{R}, \mathcal{M}$
0.5	0	1	**2**	$\mathcal{U}, \mathcal{R}, \mathcal{M}$	0.451	0.466	**0.512**	$\mathcal{U}, \mathcal{R}, \mathcal{M}$
0.6	0	1	**2**	$\mathcal{U}, \mathcal{R}, \mathcal{M}$	0.344	0.365	**0.422**	$\mathcal{U}, \mathcal{R}, \mathcal{M}$
0.7	0	1	**2**	$\mathcal{U}, \mathcal{R}, \mathcal{M}$	0.239	0.265	**0.335**	$\mathcal{U}, \mathcal{R}, \mathcal{M}$
0.8	0	1	**2**	$\mathcal{U}, \mathcal{R}, \mathcal{M}$	0.135	0.168	**0.250**	$\mathcal{U}, \mathcal{R}, \mathcal{M}$
0.9	0	1	**2**	$\mathcal{U}, \mathcal{R}, \mathcal{M}$	0.032	0.072	**0.169**	$\mathcal{U}, \mathcal{R}, \mathcal{M}$

Table B: Wins and scores under very weak selection. The table shows the same type of data as **Tab. 2**, but again using a selection strength of $\beta = 1$ instead of $\beta = 100$. With respect to both measures, wins and scores, $\mathcal{U}$ is always ranked first. Simulations are run for $T = 10^9$ time steps.

Cost c	Self payoff			Combined score			
	$\bar{\pi}_{\mathcal{M},\mathcal{M}}$	$\bar{\pi}_{\mathcal{R},\mathcal{R}}$	$\bar{\pi}_{\mathcal{U},\mathcal{U}}$	$\mathcal{M}$	$\mathcal{R}$	$\mathcal{U}$	Ranking
0.1	0.449	**0.450**	0.443	1.339	1.341	**1.347**	$\mathcal{U}$, $\mathcal{R}$, $\mathcal{M}$
0.2	**0.397**	0.396	0.387	1.175	1.178	**1.189**	$\mathcal{U}$, $\mathcal{R}$, $\mathcal{M}$
0.3	**0.346**	0.343	0.333	1.013	1.018	**1.035**	$\mathcal{U}$, $\mathcal{R}$, $\mathcal{M}$
0.4	**0.295**	0.291	0.280	0.853	0.861	**0.886**	$\mathcal{U}$, $\mathcal{R}$, $\mathcal{M}$
0.5	**0.244**	0.241	0.229	0.695	0.707	**0.741**	$\mathcal{U}$, $\mathcal{R}$, $\mathcal{M}$
0.6	**0.194**	0.191	0.180	0.538	0.556	**0.602**	$\mathcal{U}$, $\mathcal{R}$, $\mathcal{M}$
0.7	**0.145**	0.142	0.133	0.383	0.407	**0.467**	$\mathcal{U}$, $\mathcal{R}$, $\mathcal{M}$
0.8	**0.096**	0.093	0.087	0.230	0.261	**0.337**	$\mathcal{U}$, $\mathcal{R}$, $\mathcal{M}$
0.9	**0.048**	0.046	0.043	0.080	0.119	**0.212**	$\mathcal{U}$, $\mathcal{R}$, $\mathcal{M}$

Table C: Self scores and combined scores under very weak selection. The table shows the same type of data as **Tab. 3**, but for $\beta = 1$ instead of $\beta = 100$. The space of memory-1 strategies tends to have the largest self-payoff, but unconditional strategies have the largest combined score across all cost values.Simulations are run for $T = 10^9$ time steps.

Tournament: $\beta = 10$

Cost c	$\mathcal{M}:\mathcal{R}$		$\mathcal{M}:\mathcal{U}$		$\mathcal{R}:\mathcal{U}$		$(b-c)$
	$\bar{\pi}_{\mathcal{M},\mathcal{R}}$	$\bar{\pi}_{\mathcal{R},\mathcal{M}}$	$\bar{\pi}_{\mathcal{M},\mathcal{U}}$	$\bar{\pi}_{\mathcal{U},\mathcal{M}}$	$\bar{\pi}_{\mathcal{R},\mathcal{U}}$	$\bar{\pi}_{\mathcal{U},\mathcal{R}}$	
0.1	0.441	**0.454**	0.397	**0.453**	0.406	**0.446**	0.9
0.2	0.361	**0.388**	0.307	**0.386**	0.326	**0.360**	0.8
0.3	0.288	**0.329**	0.227	**0.326**	0.256	**0.285**	0.7
0.4	0.221	**0.275**	0.157	**0.274**	0.196	**0.220**	0.6
0.5	0.160	**0.227**	0.097	**0.228**	0.144	**0.165**	0.5
0.6	0.106	**0.184**	0.047	**0.187**	0.099	**0.121**	0.4
0.7	0.058	**0.144**	0.00596	**0.151**	0.061	**0.084**	0.3
0.8	0.016	**0.109**	-0.026	**0.119**	0.030	**0.056**	0.2
0.9	-0.020	**0.077**	-0.051	**0.093**	0.005	**0.033**	0.1

Table D: Pairwise competitions under rather weak selection. The table shows the same type of data as **Tab. 1**, but using a selection strength of $\beta = 10$. Similar to the case of very weak selection, we find that lower memory always wins in a pairwise competition. Simulations are run for $T = 10^9$ time steps.

Cost c	Wins				Score			
	$\mathcal{M}$	$\mathcal{R}$	$\mathcal{U}$	Ranking	$\mathcal{M}$	$\mathcal{R}$	$\mathcal{U}$	Ranking
0.1	0	1	**2**	$\mathcal{U}, \mathcal{R}, \mathcal{M}$	0.839	0.860	**0.899**	$\mathcal{U}, \mathcal{R}, \mathcal{M}$
0.2	0	1	**2**	$\mathcal{U}, \mathcal{R}, \mathcal{M}$	0.668	0.714	**0.746**	$\mathcal{U}, \mathcal{R}, \mathcal{M}$
0.3	0	1	**2**	$\mathcal{U}, \mathcal{R}, \mathcal{M}$	0.515	0.585	**0.611**	$\mathcal{U}, \mathcal{R}, \mathcal{M}$
0.4	0	1	**2**	$\mathcal{U}, \mathcal{R}, \mathcal{M}$	0.378	0.472	**0.494**	$\mathcal{U}, \mathcal{R}, \mathcal{M}$
0.5	0	1	**2**	$\mathcal{U}, \mathcal{R}, \mathcal{M}$	0.257	0.371	**0.393**	$\mathcal{U}, \mathcal{R}, \mathcal{M}$
0.6	0	1	**2**	$\mathcal{U}, \mathcal{R}, \mathcal{M}$	0.153	0.283	**0.307**	$\mathcal{U}, \mathcal{R}, \mathcal{M}$
0.7	0	1	**2**	$\mathcal{U}, \mathcal{R}, \mathcal{M}$	0.064	0.206	**0.235**	$\mathcal{U}, \mathcal{R}, \mathcal{M}$
0.8	0	1	**2**	$\mathcal{U}, \mathcal{R}, \mathcal{M}$	-0.011	0.139	**0.175**	$\mathcal{U}, \mathcal{R}, \mathcal{M}$
0.9	0	1	**2**	$\mathcal{U}, \mathcal{R}, \mathcal{M}$	-0.071	0.082	**0.126**	$\mathcal{U}, \mathcal{R}, \mathcal{M}$

Table E: Wins and scores under rather weak selection. The table shows the same type of data as **Tab. 2**, but using a selection strength of $\beta = 10$. Similar to the case of very weak selection, $\mathcal{U}$ is always ranked first. Simulations are run for $T = 10^9$ time steps.

Cost c	Self payoff			Combined score			
	$\bar{\pi}_{\mathcal{M},\mathcal{M}}$	$\bar{\pi}_{\mathcal{R},\mathcal{R}}$	$\bar{\pi}_{\mathcal{U},\mathcal{U}}$	$\mathcal{M}$	$\mathcal{R}$	$\mathcal{U}$	Ranking
0.1	0.451	**0.452**	0.376	1.289	**1.312**	1.275	$\mathcal{R}, \mathcal{M}, \mathcal{U}$
0.2	**0.381**	0.376	0.275	1.049	**1.089**	1.021	$\mathcal{R}, \mathcal{M}, \mathcal{U}$
0.3	**0.317**	0.307	0.197	0.832	**0.892**	0.808	$\mathcal{R}, \mathcal{M}, \mathcal{U}$
0.4	**0.258**	0.245	0.139	0.636	**0.717**	0.633	$\mathcal{R}, \mathcal{M}, \mathcal{U}$
0.5	**0.204**	0.189	0.097	0.461	**0.561**	0.490	$\mathcal{R}, \mathcal{M}, \mathcal{U}$
0.6	**0.154**	0.140	0.066	0.307	**0.422**	0.373	$\mathcal{R}, \mathcal{M}, \mathcal{U}$
0.7	**0.109**	0.096	0.043	0.172	**0.302**	0.278	$\mathcal{R}, \mathcal{M}, \mathcal{U}$
0.8	**0.068**	0.058	0.025	0.058	0.197	**0.200**	$\mathcal{U}, \mathcal{R}, \mathcal{M}$
0.9	**0.032**	0.026	0.011	-0.039	0.108	**0.137**	$\mathcal{U}, \mathcal{R}, \mathcal{M}$

Table F: Self scores and combined scores under rather weak selection. The table shows the same type of data as **Tab. 3**, but with $\beta = 10$. Again, the space of memory-1 strategies tends to have the largest self-payoff, but reactive and unconditional strategies have the largest combined score. Simulations are run for $T = 10^9$ time steps.

Tournament: $\beta = 1000$

Cost c	$\mathcal{M}:\mathcal{R}$		$\mathcal{M}:\mathcal{U}$		$\mathcal{R}:\mathcal{U}$		$(b-c)$
	$\bar{\pi}_{\mathcal{M},\mathcal{R}}$	$\bar{\pi}_{\mathcal{R},\mathcal{M}}$	$\bar{\pi}_{\mathcal{M},\mathcal{U}}$	$\bar{\pi}_{\mathcal{U},\mathcal{M}}$	$\bar{\pi}_{\mathcal{R},\mathcal{U}}$	$\bar{\pi}_{\mathcal{U},\mathcal{R}}$	
0.1	**0.785**	0.782	**0.273**	0.158	**0.244**	0.123	0.9
0.2	0.488	**0.494**	**0.139**	0.093	**0.112**	0.063	0.8
0.3	0.250	**0.272**	**0.069**	0.055	**0.054**	0.032	0.7
0.4	0.124	**0.153**	**0.033**	0.033	**0.026**	0.017	0.6
0.5	0.058	**0.085**	0.015	**0.019**	**0.013**	0.009	0.5
0.6	0.024	**0.044**	0.0059	**0.011**	**0.006**	0.005	0.4
0.7	0.007	**0.021**	0.002	**0.007**	**0.003**	0.002	0.3
0.8	0.001	**0.009**	-0.00008	**0.004**	**0.001**	0.001	0.2
0.9	-0.001	**0.004**	-0.001	**0.002**	**0.0004**	0.0003	0.1

Table G: Pairwise competitions under very strong selection. The table shows the same type of data as **Tab. 1**, but using a selection strength of $\beta = 1000$. Reactive players always win against unconditional players. They also win against memory-1 players when $c \geq 0.2$.Simulations are run for $T = 10^9$ time steps.

Cost c	Wins				Score			
	$\mathcal{M}$	$\mathcal{R}$	$\mathcal{U}$	Ranking	$\mathcal{M}$	$\mathcal{R}$	$\mathcal{U}$	Ranking
0.1	**2**	1	0	$\mathcal{M},\mathcal{R},\mathcal{U}$	**1.060**	1.030	0.281	$\mathcal{M},\mathcal{R},\mathcal{U}$
0.2	1	**2**	0	$\mathcal{R},\mathcal{M},\mathcal{U}$	**0.626**	0.606	0.156	$\mathcal{M},\mathcal{R},\mathcal{U}$
0.3	1	**2**	0	$\mathcal{R},\mathcal{M},\mathcal{U}$	0.319	**0.325**	0.087	$\mathcal{R},\mathcal{M},\mathcal{U}$
0.4	1	**2**	0	$\mathcal{R},\mathcal{M},\mathcal{U}$	0.157	**0.179**	0.049	$\mathcal{R},\mathcal{M},\mathcal{U}$
0.5	0	**2**	1	$\mathcal{R},\mathcal{U},\mathcal{M}$	0.073	**0.097**	0.028	$\mathcal{R},\mathcal{M},\mathcal{U}$
0.6	0	**2**	1	$\mathcal{R},\mathcal{U},\mathcal{M}$	0.030	**0.050**	0.016	$\mathcal{R},\mathcal{M},\mathcal{U}$
0.7	0	**2**	1	$\mathcal{R},\mathcal{U},\mathcal{M}$	0.009	**0.024**	0.009	$\mathcal{R},\mathcal{U},\mathcal{M}$
0.8	0	**2**	1	$\mathcal{R},\mathcal{U},\mathcal{M}$	0.001	**0.010**	0.005	$\mathcal{R},\mathcal{U},\mathcal{M}$
0.9	0	**2**	1	$\mathcal{R},\mathcal{U},\mathcal{M}$	-0.002	**0.004**	0.002	$\mathcal{R},\mathcal{U},\mathcal{M}$

Table H: Wins and scores under very strong selection. The table shows the same type of data as **Tab. 2**, but using a selection strength of $\beta = 1000$. In most cases, reactive strategies rank first with respect to both, wins and score.Simulations are run for $T = 10^9$ time steps.

Cost c	Self payoff			Combined score			
	$\bar{\pi}_{\mathcal{M},\mathcal{M}}$	$\bar{\pi}_{\mathcal{R},\mathcal{R}}$	$\bar{\pi}_{\mathcal{U},\mathcal{U}}$	$\mathcal{M}$	$\mathcal{R}$	$\mathcal{U}$	Ranking
0.1	**0.854**	0.735	0.009	**1.912**	1.762	0.290	$\mathcal{M}, \mathcal{R}, \mathcal{U}$
0.2	**0.668**	0.449	0.004	**1.294**	1.055	0.160	$\mathcal{M}, \mathcal{R}, \mathcal{U}$
0.3	**0.434**	0.246	0.002	**0.753**	0.5714	0.090	$\mathcal{M}, \mathcal{R}, \mathcal{U}$
0.4	**0.225**	0.127	0.002	**0.382**	0.305	0.051	$\mathcal{M}, \mathcal{R}, \mathcal{U}$
0.5	**0.105**	0.059	0.001	**0.178**	0.156	0.029	$\mathcal{M}, \mathcal{R}, \mathcal{U}$
0.6	**0.053**	0.025	0.001	**0.082**	0.075	0.016	$\mathcal{M}, \mathcal{R}, \mathcal{U}$
0.7	**0.024**	0.009	0.0004	**0.033**	0.033	0.009	$\mathcal{M}, \mathcal{R}, \mathcal{U}$
0.8	**0.010**	0.003	0.0003	0.011	**0.013**	0.005	$\mathcal{R}, \mathcal{M}, \mathcal{U}$
0.9	**0.003**	0.0007	0.0001	0.001	**0.005**	0.002	$\mathcal{R}, \mathcal{U}, \mathcal{M}$

Table I: Self scores and combined scores under very strong selection. The table shows the same type of data as **Tab. 3**, but with $\beta = 1000$. Memory-1 strategies have the largest self payoff. They also have the largest combined score provided $c \leq 0.7$.Simulations are run for $T = 10^9$ time steps.

Model variations and extensions: Tables for $\beta = 100$

Baseline model

Cost c	Self payoff			Combined score				$(b-c)$
	$\bar{\pi}_{\mathcal{M},\mathcal{M}}$	$\bar{\pi}_{\mathcal{R},\mathcal{R}}$	$\bar{\pi}_{\mathcal{U},\mathcal{U}}$	$\mathcal{M}$	$\mathcal{R}$	$\mathcal{U}$	Ranking	
0.1	**0.664**	0.538	0.090	**1.593**	1.478	0.613	$\mathcal{M}, \mathcal{R}, \mathcal{U}$	0.9
0.2	**0.478**	0.382	0.040	**1.095**	1.030	0.354	$\mathcal{M}, \mathcal{R}, \mathcal{U}$	0.8
0.3	**0.322**	0.267	0.023	**0.717**	0.711	0.226	$\mathcal{M}, \mathcal{R}, \mathcal{U}$	0.7
0.4	**0.210**	0.180	0.015	0.453	**0.483**	0.146	$\mathcal{R}, \mathcal{M}, \mathcal{U}$	0.6
0.5	**0.135**	0.114	0.010	0.276	**0.316**	0.095	$\mathcal{R}, \mathcal{M}, \mathcal{U}$	0.5
0.6	**0.084**	0.067	0.0067	0.1582	**0.196**	0.061	$\mathcal{R}, \mathcal{M}, \mathcal{U}$	0.4
0.7	**0.049**	0.035	0.0043	0.0806	**0.113**	0.038	$\mathcal{R}, \mathcal{M}, \mathcal{U}$	0.3
0.8	**0.024**	0.016	0.0025	0.0321	**0.059**	0.022	$\mathcal{R}, \mathcal{M}, \mathcal{U}$	0.2
0.9	**0.009**	0.005	0.001	0.004	**0.026**	0.011	$\mathcal{R}, \mathcal{U}, \mathcal{M}$	0.1

Table J: Self scores and combined scores in pairwise tournaments. For ease of comparison with the data below, we present the data from **Tab. 3**once more. Across all cooperation costs, we find that memory-1 strategies yield the largest self payoff. They also achieve the largest combined score if $c \leq 0.3$; otherwise, for $c \geq 0.4$, reactive strategies succeed. Simulations are run for $T = 10^9$ time steps.

Sampling from a U-shaped distribution

Cost c	Self payoff			Combined score			
	$\bar{\pi}_{\mathcal{M},\mathcal{M}}$	$\bar{\pi}_{\mathcal{R},\mathcal{R}}$	$\bar{\pi}_{\mathcal{U},\mathcal{U}}$	$\mathcal{M}$	$\mathcal{R}$	$\mathcal{U}$	Ranking
0.1	**0.657**	0.535	0.046	**1.576**	1.460	0.499	$\mathcal{M}, \mathcal{R}, \mathcal{U}$
0.2	**0.503**	0.399	0.020	**1.148**	1.066	0.311	$\mathcal{M}, \mathcal{R}, \mathcal{U}$
0.3	**0.372**	0.296	0.012	**0.820**	0.788	0.215	$\mathcal{M}, \mathcal{R}, \mathcal{U}$
0.4	**0.262**	0.216	0.008	0.563	**0.581**	0.151	$\mathcal{R}, \mathcal{M}, \mathcal{U}$
0.5	**0.175**	0.153	0.005	0.364	**0.421**	0.104	$\mathcal{R}, \mathcal{M}, \mathcal{U}$
0.6	**0.115**	0.102	0.003	0.223	**0.293**	0.070	$\mathcal{R}, \mathcal{M}, \mathcal{U}$
0.7	**0.071**	0.060	0.002	0.121	**0.189**	0.044	$\mathcal{R}, \mathcal{M}, \mathcal{U}$
0.8	**0.038**	0.030	0.001	0.049	**0.108**	0.025	$\mathcal{R}, \mathcal{M}, \mathcal{U}$
0.9	**0.010**	0.007	0.001	0.003	**0.050**	0.012	$\mathcal{R}, \mathcal{M}, \mathcal{U}$

Table K: Self scores and combined scores for Extension 1 under $\beta = 100$. The table shows the same type of data as **Tab. 3**, but for the model extension where we sample all strategies from a U-shaped distribution. We find that memory-1 strategies retain the largest self payoff for each value of c. They also have the largest combined score for $c < 0.4$, whereas reactive strategies win for higher values of c. Simulations are run for $T = 10^9$ time steps.

Same complexity sampling I: 4 random numbers, biasing towards boundary

Cost c	Self payoff			Combined score			
	$\bar{\pi}_{\mathcal{M},\mathcal{M}}$	$\bar{\pi}_{\mathcal{R},\mathcal{R}}$	$\bar{\pi}_{\mathcal{U},\mathcal{U}}$	$\mathcal{M}$	$\mathcal{R}$	$\mathcal{U}$	Ranking
0.1	**0.664**	0.479	0.051	**1.540**	1.337	0.565	$\mathcal{M}, \mathcal{R}, \mathcal{U}$
0.2	**0.477**	0.337	0.029	**1.047**	0.922	0.362	$\mathcal{M}, \mathcal{R}, \mathcal{U}$
0.3	**0.322**	0.240	0.019	**0.668**	0.652	0.240	$\mathcal{M}, \mathcal{R}, \mathcal{U}$
0.4	**0.210**	0.172	0.013	0.404	**0.472**	0.161	$\mathcal{R}, \mathcal{M}, \mathcal{U}$
0.5	**0.135**	0.123	0.008	0.233	**0.345**	0.108	$\mathcal{R}, \mathcal{M}, \mathcal{U}$
0.6	**0.084**	0.081	0.006	0.121	**0.241**	0.072	$\mathcal{R}, \mathcal{M}, \mathcal{U}$
0.7	**0.048**	0.047	0.004	0.050	**0.154**	0.047	$\mathcal{R}, \mathcal{M}, \mathcal{U}$
0.8	**0.024**	0.022	0.002	0.007	**0.088**	0.029	$\mathcal{R}, \mathcal{M}, \mathcal{U}$
0.9	**0.009**	0.007	0.001	-0.015	**0.042**	0.017	$\mathcal{R}, \mathcal{M}, \mathcal{U}$

Table L: Self scores and combined scores for Extension 2 under $\beta = 100$. The table shows the same type of data as **Tab. 3**, but for the model extension where we sample strategies by drawing four values each, and then choosing the value(s) closest to the boundary for reactive and unconditional strategies. This biases strategies away from 0.5. We find that memory-1 strategies retain the largest self payoff for each value of c. They also have the largest combined score for $c < 0.3$, whereas reactive strategies win for higher values of c. Simulations are run for $T = 10^9$ time steps.

Same complexity sampling II: 4 random numbers, averaging

Cost c	Self payoff			Combined score			
	$\bar{\pi}_{\mathcal{M},\mathcal{M}}$	$\bar{\pi}_{\mathcal{R},\mathcal{R}}$	$\bar{\pi}_{\mathcal{U},\mathcal{U}}$	$\mathcal{M}$	$\mathcal{R}$	$\mathcal{U}$	Ranking
0.1	**0.664**	0.481	0.280	**1.587**	1.400	0.823	$\mathcal{M}, \mathcal{R}, \mathcal{U}$
0.2	**0.478**	0.348	0.155	**1.156**	1.015	0.417	$\mathcal{M}, \mathcal{R}, \mathcal{U}$
0.3	**0.322**	0.255	0.093	**0.820**	0.727	0.211	$\mathcal{M}, \mathcal{R}, \mathcal{U}$
0.4	**0.210**	0.177	0.060	**0.576**	0.499	0.097	$\mathcal{M}, \mathcal{R}, \mathcal{U}$
0.5	**0.135**	0.113	0.040	**0.405**	0.322	0.029	$\mathcal{M}, \mathcal{R}, \mathcal{U}$
0.6	**0.084**	0.066	0.027	**0.283**	0.195	-0.014	$\mathcal{M}, \mathcal{R}, \mathcal{U}$
0.7	**0.048**	0.036	0.017	**0.198**	0.113	-0.042	$\mathcal{M}, \mathcal{R}, \mathcal{U}$
0.8	**0.024**	0.017	0.010	**0.139**	0.062	-0.061	$\mathcal{M}, \mathcal{R}, \mathcal{U}$
0.9	**0.009**	0.007	0.004	**0.098**	0.030	-0.072	$\mathcal{M}, \mathcal{R}, \mathcal{U}$

Table M: Self scores and combined scores for Extension 3 under $\beta = 100$. The table shows the same type of data as **Tab. 3**, but for the model extension where we sample four values for each strategy space, and average them to construct lower memory strategies. Reactive strategies are composed by averaging two values each to get a tuple, whereas unconditional strategies are constructed by averaging all four sampled values to get the player's cooperation probability. Memory-1 strategies have the largest self payoff. They also have the largest combined score for all values of c. Simulations are run for $T = 10^9$ time steps.

Strategy imitation with inference

Cost c	Self payoff			Combined score			
	$\bar{\pi}_{\mathcal{M},\mathcal{M}}$	$\bar{\pi}_{\mathcal{R},\mathcal{R}}$	$\bar{\pi}_{\mathcal{U},\mathcal{U}}$	$\mathcal{M}$	$\mathcal{R}$	$\mathcal{U}$	Ranking
0.1	**0.719**	0.554	0.090	**1.587**	1.445	0.585	$\mathcal{M}, \mathcal{R}, \mathcal{U}$
0.2	**0.550**	0.390	0.040	**1.097**	0.981	0.325	$\mathcal{M}, \mathcal{R}, \mathcal{U}$
0.3	**0.383**	0.258	0.024	**0.720**	0.652	0.200	$\mathcal{M}, \mathcal{R}, \mathcal{U}$
0.4	**0.225**	0.167	0.015	**0.426**	0.419	0.126	$\mathcal{M}, \mathcal{R}, \mathcal{U}$
0.5	**0.127**	0.101	0.010	0.242	**0.260**	0.079	$\mathcal{R}, \mathcal{M}, \mathcal{U}$
0.6	**0.076**	0.057	0.007	0.138	**0.151**	0.049	$\mathcal{R}, \mathcal{M}, \mathcal{U}$
0.7	**0.040**	0.030	0.004	0.070	**0.081**	0.030	$\mathcal{R}, \mathcal{M}, \mathcal{U}$
0.8	**0.019**	0.014	0.003	0.031	**0.039**	0.016	$\mathcal{R}, \mathcal{M}, \mathcal{U}$
0.9	**0.007**	0.004	0.001	0.010	**0.014**	0.008	$\mathcal{R}, \mathcal{M}, \mathcal{U}$

Table N: Self scores and combined scores for Extension 4 under $\beta = 100$. The table shows the same type of data as **Tab. 3**, but for the model extension where high memory players imitate their co-player's strategy with probability $\alpha = 0.05$, whereas with the same probability low-memory players infer their co-player's effective memory strategy. Memory-1 strategies still have the largest self payoff. They also have the largest combined score for $c > 0.4$, whereas reactive strategies win for higher values of c. We note however that compared to **Tab. 3**, the competition is tighter. Simulations are run for $T = 10^9$ time steps.

Modifying the strategy updating process: Search for favorable strategies

Cost c	Self payoff			Combined score			
	$\bar{\pi}_{\mathcal{M},\mathcal{M}}$	$\bar{\pi}_{\mathcal{R},\mathcal{R}}$	$\bar{\pi}_{\mathcal{U},\mathcal{U}}$	$\mathcal{M}$	$\mathcal{R}$	$\mathcal{U}$	Ranking
0.1	**0.485**	0.450	0.143	**1.33**	1.32	0.797	$\mathcal{M}, \mathcal{R}, \mathcal{U}$
0.2	**0.372**	0.349	0.064	1.04	**1.05**	0.481	$\mathcal{R}, \mathcal{M}, \mathcal{U}$
0.3	**0.286**	0.271	0.037	0.803	**0.830**	0.314	$\mathcal{R}, \mathcal{M}, \mathcal{U}$
0.4	**0.216**	0.206	0.024	0.604	**0.642**	0.209	$\mathcal{R}, \mathcal{M}, \mathcal{U}$
0.5	**0.160**	0.150	0.016	0.437	**0.481**	0.136	$\mathcal{R}, \mathcal{M}, \mathcal{U}$
0.6	**0.113**	0.103	0.011	0.297	**0.343**	0.083	$\mathcal{R}, \mathcal{M}, \mathcal{U}$
0.7	**0.074**	0.064	0.007	0.183	**0.226**	0.044	$\mathcal{R}, \mathcal{M}, \mathcal{U}$
0.8	**0.043**	0.033	0.004	0.093	**0.131**	0.018	$\mathcal{R}, \mathcal{M}, \mathcal{U}$
0.9	**0.018**	0.012	0.002	0.028	**0.059**	0.0032	$\mathcal{R}, \mathcal{M}, \mathcal{U}$

Table O: Self scores and combined scores when players can actively search for better strategies under $\beta = 100$. The table shows the same type of data as **Tab. 3**, but for the model extension where players are allowed to search for a new strategy until the mutant is accepted. We find that Memory-1 strategies still have the largest self payoff. They also have the largest combined score for $c < 0.2$, whereas reactive strategies win for higher values of c. Simulations are run for $T = 10^9$ time steps.

Baseline model for $\beta = 10$

Cost c	Self payoff			Combined score			
	$\bar{\pi}_{\mathcal{M},\mathcal{M}}$	$\bar{\pi}_{\mathcal{R},\mathcal{R}}$	$\bar{\pi}_{\mathcal{U},\mathcal{U}}$	$\mathcal{M}$	$\mathcal{R}$	$\mathcal{U}$	Ranking
0.1	0.451	**0.452**	0.376	1.289	**1.312**	1.275	$\mathcal{R}, \mathcal{M}, \mathcal{U}$
0.2	**0.381**	0.376	0.275	1.049	**1.089**	1.021	$\mathcal{R}, \mathcal{M}, \mathcal{U}$
0.3	**0.317**	0.307	0.197	0.832	**0.892**	0.808	$\mathcal{R}, \mathcal{M}, \mathcal{U}$
0.4	**0.258**	0.245	0.139	0.636	**0.717**	0.633	$\mathcal{R}, \mathcal{M}, \mathcal{U}$
0.5	**0.204**	0.189	0.097	0.461	**0.561**	0.490	$\mathcal{R}, \mathcal{M}, \mathcal{U}$
0.6	**0.154**	0.140	0.066	0.307	**0.422**	0.373	$\mathcal{R}, \mathcal{M}, \mathcal{U}$
0.7	**0.109**	0.096	0.043	0.172	**0.302**	0.278	$\mathcal{R}, \mathcal{M}, \mathcal{U}$
0.8	**0.068**	0.058	0.025	0.058	0.197	**0.200**	$\mathcal{U}, \mathcal{R}, \mathcal{M}$
0.9	**0.032**	0.026	0.011	-0.039	0.108	**0.137**	$\mathcal{U}, \mathcal{R}, \mathcal{M}$

Table P: Self scores and combined scores under rather weak selection. The table shows the same type of data as **Tab. 3**, but with $\beta = 10$. Again, the space of memory-1 strategies tends to have the largest self-payoff, but reactive and unconditional strategies have the largest combined score. Simulations are run for $T = 10^9$ time steps.

Model variations and extensions: Tables for $\beta = 10$

Sampling from a U-shaped distribution

Cost c	Self payoff			Combined score			
	$\bar{\pi}_{\mathcal{M},\mathcal{M}}$	$\bar{\pi}_{\mathcal{R},\mathcal{R}}$	$\bar{\pi}_{\mathcal{U},\mathcal{U}}$	$\mathcal{M}$	$\mathcal{R}$	$\mathcal{U}$	Ranking
0.1	**0.511**	0.496	0.358	1.403	**1.42**	1.31	$\mathcal{R}, \mathcal{M}, \mathcal{U}$
0.2	**0.422**	0.406	0.229	1.12	**1.16**	0.977	$\mathcal{R}, \mathcal{M}, \mathcal{U}$
0.3	**0.343**	0.328	0.143	0.878	**0.943**	0.719	$\mathcal{R}, \mathcal{M}, \mathcal{U}$
0.4	**0.272**	0.259	0.090	0.663	**0.753**	0.524	$\mathcal{R}, \mathcal{M}, \mathcal{U}$
0.5	**0.210**	0.197	0.057	0.476	**0.587**	0.379	$\mathcal{R}, \mathcal{M}, \mathcal{U}$
0.6	**0.155**	0.144	0.036	0.316	**0.442**	0.269	$\mathcal{R}, \mathcal{M}, \mathcal{U}$
0.7	**0.107**	0.097	0.022	0.181	**0.316**	0.186	$\mathcal{R}, \mathcal{U}, \mathcal{M}$
0.8	**0.065**	0.057	0.013	0.071	**0.208**	0.123	$\mathcal{R}, \mathcal{U}, \mathcal{M}$
0.9	**0.029**	0.025	0.006	-0.018	**0.119**	0.076	$\mathcal{R}, \mathcal{U}, \mathcal{M}$

Table Q: Self scores and combined scores for Extension 1 under $\beta = 10$. The table shows the same type of data as **Tab. 3**, but for the model extension where we sample all strategies from a U-shaped distribution. We find that memory-1 strategies retain the largest self payoff for each value of c. Meanwhile, reactive strategies have the largest combined score for all values of c. Simulations are run for $T = 10^9$ time steps.

Same complexity sampling I: 4 random numbers, biasing towards boundary

Cost c	Self payoff			Combined score			
	$\bar{\pi}_{\mathcal{M},\mathcal{M}}$	$\bar{\pi}_{\mathcal{R},\mathcal{R}}$	$\bar{\pi}_{\mathcal{U},\mathcal{U}}$	$\mathcal{M}$	$\mathcal{R}$	$\mathcal{U}$	Ranking
0.1	0.451	**0.463**	0.307	1.26	**1.32**	1.22	$\mathcal{R}, \mathcal{M}, \mathcal{U}$
0.2	**0.381**	0.378	0.178	0.986	**1.08**	0.918	$\mathcal{R}, \mathcal{M}, \mathcal{U}$
0.3	**0.317**	0.304	0.104	0.743	**0.877**	0.702	$\mathcal{R}, \mathcal{M}, \mathcal{U}$
0.4	**0.258**	0.240	0.065	0.530	**0.705**	0.550	$\mathcal{R}, \mathcal{U}, \mathcal{M}$
0.5	**0.204**	0.183	0.044	0.345	**0.556**	0.437	$\mathcal{R}, \mathcal{U}, \mathcal{M}$
0.6	**0.154**	0.133	0.030	0.188	**0.426**	0.350	$\mathcal{R}, \mathcal{U}, \mathcal{M}$
0.7	**0.109**	0.090	0.021	0.058	**0.312**	0.279	$\mathcal{R}, \mathcal{U}, \mathcal{M}$
0.8	**0.068**	0.053	0.013	-0.048	0.213	**0.222**	$\mathcal{U}, \mathcal{R}, \mathcal{M}$
0.9	**0.032**	0.024	0.006	-0.132	0.130	**0.175**	$\mathcal{U}, \mathcal{R}, \mathcal{M}$

Table R: Self scores and combined scores for Extension 2 under $\beta = 10$. The table shows the same type of data as **Tab. 3**, but for the model extension where we sample strategies by drawing four values each, and then choosing the value(s) closest to the boundary for reactive and unconditional strategies. This biases strategies away from 0.5. We find that memory-1 strategies retain the largest self payoff for $c > 0.1$. However, reactive strategies have the largest combined score for $c < 0.8$, whereas unconditional strategies win for higher values of c. Simulations are run for $T = 10^9$ time steps.

Same complexity sampling II: 4 random numbers, averaging

Cost c	Self payoff			Combined score			
	$\bar{\pi}_{\mathcal{M},\mathcal{M}}$	$\bar{\pi}_{\mathcal{R},\mathcal{R}}$	$\bar{\pi}_{\mathcal{U},\mathcal{U}}$	$\mathcal{M}$	$\mathcal{R}$	$\mathcal{U}$	Ranking
0.1	**0.451**	0.441	0.431	**1.329**	0.202	1.302	$\mathcal{M}, \mathcal{R}, \mathcal{U}$
0.2	**0.381**	0.377	0.367	**1.136**	1.120	1.100	$\mathcal{M}, \mathcal{R}, \mathcal{U}$
0.3	0.316	**0.317**	0.307	**0.959**	0.938	0.908	$\mathcal{M}, \mathcal{R}, \mathcal{U}$
0.4	0.258	**0.261**	0.251	**0.799**	0.770	0.727	$\mathcal{M}, \mathcal{R}, \mathcal{U}$
0.5	0.204	**0.208**	0.199	**0.652**	0.613	0.557	$\mathcal{M}, \mathcal{R}, \mathcal{U}$
0.6	0.154	**0.160**	0.152	**0.520**	0.468	0.399	$\mathcal{M}, \mathcal{R}, \mathcal{U}$
0.7	0.109	**0.114**	0.108	**0.401**	0.334	0.252	$\mathcal{M}, \mathcal{R}, \mathcal{U}$
0.8	0.068	**0.073**	0.069	**0.296**	0.211	0.116	$\mathcal{M}, \mathcal{R}, \mathcal{U}$
0.9	0.032	**0.034**	0.033	**0.202**	0.101	-0.009	$\mathcal{M}, \mathcal{R}, \mathcal{U}$

Table S: Self scores and combined scores for Extension 3 under $\beta = 10$. The table shows the same type of data as **Tab. 3**, but for the model extension where we sample four values for each strategy space, and use averaging to construct lower memory strategies. Reactive strategies have the largest self payoff for most values of c. However, memory-1 has the largest combined score for all values of c. Simulations are run for $T = 10^9$ time steps.

Strategy imitation with inference

Cost c	Self payoff			Combined score			
	$\bar{\pi}_{\mathcal{M},\mathcal{M}}$	$\bar{\pi}_{\mathcal{R},\mathcal{R}}$	$\bar{\pi}_{\mathcal{U},\mathcal{U}}$	$\mathcal{M}$	$\mathcal{R}$	$\mathcal{U}$	Ranking
0.1	**0.457**	0.454	0.376	1.30	**1.31**	1.25	$\mathcal{R}, \mathcal{M}, \mathcal{U}$
0.2	**0.383**	0.375	0.274	1.06	**1.08**	0.995	$\mathcal{R}, \mathcal{M}, \mathcal{U}$
0.3	**0.319**	0.306	0.197	0.843	**0.883**	0.779	$\mathcal{R}, \mathcal{M}, \mathcal{U}$
0.4	**0.259**	0.243	0.139	0.650	**0.703**	0.601	$\mathcal{R}, \mathcal{M}, \mathcal{U}$
0.5	**0.204**	0.187	0.097	0.480	**0.542**	0.456	$\mathcal{R}, \mathcal{M}, \mathcal{U}$
0.6	**0.153**	0.137	0.065	0.330	**0.402**	0.337	$\mathcal{R}, \mathcal{M}, \mathcal{U}$
0.7	**0.108**	0.094	0.043	0.203	**0.282**	0.242	$\mathcal{R}, \mathcal{U}, \mathcal{M}$
0.8	**0.067**	0.058	0.025	0.092	**0.181**	0.165	$\mathcal{R}, \mathcal{U}, \mathcal{M}$
0.9	**0.039**	0.027	0.011	0.023	0.002	**0.103**	$\mathcal{U}, \mathcal{R}, \mathcal{M}$

Table T: Self scores and combined scores for Extension 4 under $\beta = 10$. The table shows the same type of data as **Tab. 3**, but for the model extension where high memory players imitate their co-player's strategy with probability $\alpha = 0.05$, whereas with the same probability low-memory players infer their co-player's effective memory strategy. Memory-1 strategies still have the largest self payoff. However, reactive strategies have the largest combined score for $c < 0.9$. Simulations are run for $T = 10^9$ time steps.

Modifying the strategy updating process: Search for favorable strategies

Cost c	Self payoff			Combined score			
	$\bar{\pi}_{\mathcal{M},\mathcal{M}}$	$\bar{\pi}_{\mathcal{R},\mathcal{R}}$	$\bar{\pi}_{\mathcal{U},\mathcal{U}}$	$\mathcal{M}$	$\mathcal{R}$	$\mathcal{U}$	Ranking
0.1	0.448	**0.449**	0.412	1.30	1.32	**1.33**	$\mathcal{U}, \mathcal{R}, \mathcal{M}$
0.2	**0.388**	0.382	0.332	1.09	1.11	**1.13**	$\mathcal{U}, \mathcal{R}, \mathcal{M}$
0.3	**0.330**	0.321	0.259	0.892	0.928	**0.943**	$\mathcal{U}, \mathcal{R}, \mathcal{M}$
0.4	**0.275**	0.263	0.196	0.703	0.758	**0.780**	$\mathcal{U}, \mathcal{R}, \mathcal{M}$
0.5	**0.223**	0.210	0.143	0.526	0.603	**0.636**	$\mathcal{U}, \mathcal{R}, \mathcal{M}$
0.6	**0.173**	0.160	0.101	0.359	0.462	**0.511**	$\mathcal{U}, \mathcal{R}, \mathcal{M}$
0.7	**0.126**	0.114	0.067	0.205	0.333	**0.403**	$\mathcal{U}, \mathcal{R}, \mathcal{M}$
0.8	**0.081**	0.072	0.039	0.064	0.217	**0.310**	$\mathcal{U}, \mathcal{R}, \mathcal{M}$
0.9	**0.039**	0.034	0.018	-0.065	0.112	**0.230**	$\mathcal{U}, \mathcal{R}, \mathcal{M}$

Table U: Self scores and combined scores for Extension 5 under $\beta = 10$. The table shows the same type of data as **Tab. 3**, but for the model extension where players are allowed to search for a new strategy until the mutant is accepted. Memory-1 strategies still have the largest self payoff except for $c = 0.1$. However, unconditional strategies have the largest combined score for all values of c. Simulations are run for $T = 10^9$ time steps.
