# Supplementary material for: Direct reciprocity between individuals that use different strategy spaces
Source: PLoS Comput Biol. 2022 Jun 14;18(6):e1010149. doi: 10.1371/journal.pcbi.1010149 (PMC9197081; doi:10.1371/journal.pcbi.1010149)
Supplement: Attachment — Submitted filename: plosReview.pdf [file pcbi.1010149.s012.pdf]

Contribution:
This paper studies direct reciprocity through repeated donation games and has interesting findings on the behavioral strategies. It explores the role of memory capacity on cooperation. It touches base on an important assumption in the field of repeated game theory that history is perfectly memorized. In the main result, they identify three types of strategies spaces that differ in memory demands and found that smaller strategy spaces dominate larger ones.

Overall I found the topic interesting but the paper itself needs to be a lot more clear in order to fit the interest to a broader audience. It needs to be explained in a much clearer way in order for it to be published in any journal. I feel it will be hopeless for someone to understand this manuscript if they don't read other articles on this set of notation. It is not that the math is complicated but just a matter of explanation. The authors must be aware that this journal is biological communication, most of the readers will have zero knowledge of game theory terminologies.

I am afraid I cannot accept the paper unless the manuscript can be improved.
My specific suggestions are as follows:

> The introduction part in my opinion is a bit too narrowed that it only touch base on this small circle of literature within memory-based games. As a person who doesn't belong to this literature, I don't get it immediately why we need to study this domain. Though personally, I think it's important. I just didn't get it from the writing.
>
> The author needs to say why this toy model they are simulating is important and how it maps to life-like scenarios. This is important as we can't just simulate something fun and make ourselves enthusiastic.
>
> The basic structure of the game needs to be better visualized. It can be easily done through a table. I found figure 1 is even less helpful than the word (i saw they have a clear matrix in the dynamic part).
>
> Starting from line 100, I found the notation very confusing and when a notation comes out for the first time, the authors are not explaining them clearly. One example can be, in line 103, "we refer to … set of strategies…", here you need to explain the dimension of a strategy in mathematical terms along with its dimension. You can't let readers infer according to your game structures. Where is the notation of history at this point if we are talking about strategies in a repeated game? Overall, I think the math doesn't talk to intuition.
>
> The authors need to specify what limitation the PD game causes as it is a very specific type of cooperation that the cooperative strategy is not Nash equilibrium. If possible, I think authors can at least discuss what they think will happen in other domains of repeated games, not only PD.
