# Supplementary material for: Direct reciprocity between individuals that use different strategy spaces
Source: PLoS Comput Biol. 2022 Jun 14;18(6):e1010149. doi: 10.1371/journal.pcbi.1010149 (PMC9197081; doi:10.1371/journal.pcbi.1010149)
Supplement: Attachment — Submitted filename: review_response.pdf [file pcbi.1010149.s013.pdf]

We would like to thank the editor and the three referees for their efforts. The feedback was very helpful and constructive. We have addressed all comments. We believe that the suggested changes have improved the clarity of our manuscript and the impact of our results substantially.
Please find our detailed response below.

Editor Comments:

Your manuscript has been evaluated by 3 reviewers with expertise in, respectively, evolutionary biology, game theory, and cognitive and social psychology. While the reviewers find your work relevant and interesting, they ask for better clarification of the game and model, and the exploration or discussion of alternative possibilities.

Reply: *Thanks for this positive and constructive feedback. We have amended our manuscript accordingly. In particular, we have added several model variations and extensions of our baseline model, and we have rewritten several paragraphs to further enhance clarity. We present the new model variations in the form of new figures (**Figs. 5, S8, S9, S10**), new data tables (**Tables S11-S21**), and have added a corresponding new section to the main text.*

Reviewer #1:

Schmid and colleagues studied repeated interactions between agents with different strategy spaces that required different memory capacity: unconditional players who have no memory, reactive players who responded to the last move of the co-player, and memory-1 (M1) players who consider the last moves of both parties. Combining numerical calculation and simulation, they conducted careful comparisons on the dominance of different types of agents and examined the dynamics at two scales: (i) when the strategy space of the agents was fixed but the strategies were allowed to evolve (tournaments); (ii) when the strategy space also evolved across time based on the payoff of each type of strategies (evolutionary dynamics). Their results point to an interesting “memory dilemma”: the society will be better off if all players adopt M1 strategies, while the sophisticated strategies can only dominate more naive ones under few conditions.

Overall, the research question is important, the analyses are carefully conducted, and the paper is clearly written.

Reply: *We are very grateful for this encouraging feedback!*

However, in my opinion, the memory dilemma demonstrated by the authors would have more significant implications if they provide more results or discussions on the specificity, robustness, or generality of the memory dilemma. Although the authors have already discussed the commonality of the memory dilemma in the parameter space for both tournaments and evolutionary dynamics, these analyses were based on the same assumptions including how agents change their strategies within their corresponding spaces.

Reply*: Thank you for pointing out this very valid issue. Our baseline model focuses on a basic setup with standard assumptions. In this way, we can illustrate the fundamental process and results in a straightforward manner. We agree however that our approach only illustrates a somewhat restricted range of scenarios due to the assumptions made. We have therefore*

extended the manuscript by discussing different variations of this baseline. See below for details of the changes.

Specifically, it is not clear whether the failure of M1 players is caused by the memory capacity itself (i.e., the sophistication of strategies or the contingency of strategies on the interactive histories) or by the inefficient search of the strategy space (associated with the size of the strategy space or the efficiency of the search algorithm) given the assumptions of how strategies evolve. This alternative explanation is consistent with the speculation of the authors about the possible reason why M1 strategies fails — it is relatively hard for M1 players to come up with extreme behavior, which prevent them from switching quickly to defect when cooperation is costly or the selection is weak. I thus have three main questions:

1. Does the failure specific to strategy space that requires higher memory capacity or general to strategy space that is large in size? Alternatively, do reactive/unconditional players still outperform M1 players if the sizes of their strategy spaces are matched? Although higher memory capacity often leads to larger size of the strategy space, these two factors can still be dissociated. For example, given the memory capacity, the size of the strategy space can still vary (e.g., by sampling different numbers of strategies to construct subsets of the entire space).

***Reply**: This is an excellent question. While it is indeed often assumed in the literature that complexity/size of the strategy space and memory size are directly correlated, exploring the effect of memory capacity on its own is very relevant to our research question. It can test the robustness of our results and the underlying mechanism. We therefore completely agree that analyzing the setup suggested by the reviewer is a very valuable extension of our framework. To address this, we now include results on two variations of our baseline model. For these variations, we disentangle memory capacity from the size of the strategy space, by doing what we call same-complexity sampling:*

In the first variation, we keep the number of entries for each strategy space fixed across all conditions (memory-1, reactive, and unconditional). Specifically, we sample four random numbers (p_1,p_2,p_3,p_4) for each strategy space.

- *If the player uses memory-1, the strategy (p_{CC},p_{CD},p_{DC},p_{DD}) is simply composed of these four values.*
- *If the player uses a reactive strategy, the player constructs its strategy (p,q) from the numbers (p_1,p_2,p_3,p_4) in the following way: For the first entry p, the player takes the average of the first two numbers p_1,p_2. For the second entry q, the, player takes the average of p_3,p_4.*
- *If the player chooses an unconditional strategy, the player's cooperation probability is simply the average of all four numbers p_1,p_2,p_3,p_4.*

This sampling scheme ensures that the size of the sampled strategy space is independent from the player's memory capacity.

When we explore this setup, we find that the memory dilemma vanishes for all values of c and for both weak (β=10) and strong (β=100) selection. Memory-1 players now outperform lower memory players both in terms of having a higher self-payoff, a higher "combined score", as well as being the Nash equilibrium of the evolutionary process. This adds to the overall picture that has presented itself previously: when lower memory players are no longer able to efficiently instantiate extreme behaviors, they lose their ability to win against higher memory strategies.

For the second variant of same-complexity sampling, we again pick four random numbers for each strategy space. However, to construct reactive strategies of the form (p,q) in this modification, we use a different method. For the value of p, the player picks the more extreme value out of the two numbers p_1 and p_2 (i.e., the number that is closer to the boundary of the interval

[0,1]). For the value of q, the player takes the more extreme value out of the two numbers p_3 and p_4. For unconditional strategies, the player chooses the single most extreme value out of the four numbers p_1,p_2,p_3,p_4. In contrast to the first variation, we observe that this variation still gives rise to memory dilemmas for both weak (β=10) and strong (β=100) selection.

Both variations support our previous claim that the memory dilemma arises because the strategy spaces differ in how easily they generate extreme behaviors.

***Changes:** Our manuscript now includes the results on the two variations of the baseline framework as described above. On one hand, we visualize the data for strong selection in the new figures **Figs. S8** (where we consider a fixed value of cost c) and **S9** (where we look at the full range of cost values). In Tables **S12, S18, S13** and **S19** we additionally present the data resulting from pairwise tournaments under weak and strong selection in the framework of these two model variations, in a manner corresponding to Table 3 in the main text.*
We have added a new subsection on model extensions to the Results section, where we include a detailed description of these two variations.

2. What if agents are allowed to use other algorithms that are potentially more efficient to search the strategy space, compared with the current algorithm that randomly select a new strategy and adopt it with a certain probability based on the payoffs? For example, an algorithm that reduces the probability of sampling from a local sub-space, if a strategy in it generates a very low payoff.

***Reply**: This is a great proposal. We agree that it would be extremely interesting to look at a model where individuals adapt the way how they sample within their strategy space. For example, if strategies in one particular subspace tend to be less successful, players may learn to sample from this subspace less often. However, we noted that fully incorporating such a model extension is non-trivial. For example, even if a certain subspace is disadvantageous at one point in time, it may become advantageous again at a later point (for example, once the opponent has switched to a different strategy). So in our opinion, a fully satisfactory model would have to make sure that even those subspaces that were less successful in the past are not completely abandoned. We have no doubt that such a model can be devised. However, it would require a number of additional parameters, with fine-tuned parameter values. We believe such a model is sufficiently complex (and interesting) to warrant an entire paper on its own.*

However, after careful consideration, we devised two variants of the suggested addition that we think capture the essentials of the idea behind it.

First, we have sampled new strategies from a U-shaped distribution instead of a uniform one. This means that players' new strategies tend to have parameters close to the boundary, making it slightly easier to find fully defecting or cooperating strategies. We find that this has only a small effect on results, both for β=100 and β=10; the memory dilemma is weakened for a few values of cost c. These results are consistent if we compare with the outcome of the model variation discussed above, where we bias only lower-memory strategies towards the boundary. If the sampling from all memory spaces places more weight on extreme behaviors, the lower-memory spaces retain but not expand their advantage of finding the most extreme behaviors the most quickly.

Second, and more substantial, we have explored a modification of our sampling strategy. According to this modification, players search for a new strategy until they have found a better one (instead of only getting one shot per round at revising their strategy). This automatically

implies that “bad” strategies are discarded without negatively affecting a player. We find, possibly contrary to expectations, that this modification in fact makes the memory dilemma stronger for both strong and weak selection. Additionally, self-payoffs are decreased. It appears that as higher memory players search for strategies that are "better", they are more likely to only find strategies that are incrementally better. In contrast, if lower memory finds a better strategy, it is more likely to be more extreme, leading to a stronger difference in payoffs.

***Changes**: We now include the results of the two extensions/modifications discussed above in three forms. First, we visualize results on the U-shaped distribution sampling in **Figs. S8 (b)** and **S9 (c)**. Second, we include new tables containing all data of both modifications for both strong and weak selection in the SI (**Tables S11, S17, S15, S21**). Finally, we discuss the implications of the two in our new subsection of the main text.*

3. Social learning is also an important way to improve the search in the strategy space. Although players with lower memory capacity cannot imitate strategies of those with higher memory capacity, it is possible for the latter to imitate the former. Do the memory dilemma still hold if M1 players imitate others’ strategies or simply copy others’ actions when others’ payoffs are higher than themselves?

***Reply**: We are grateful for this suggestion. Our introspection dynamics is a simple model of how individuals with different memory capacity might revise their strategies. However, we fully agree that social learning and imitation is a very natural alternative behavior. In fact, in evolutionary game theory, imitation processes are often taken as a standard model to describe the spread of strategies in a population. It is thus indeed very worthwhile to explore this variation of our baseline model. We now incorporate this suggestion in the following way.*

We have extended our manuscript by considering another variation. Here, with a certain probability alpha, players use social learning to revise their strategies. With the converse probability (1- alpha), players use introspection dynamics. For this model to be sensible, one needs to assume that players are able to infer their co-players’ strategies from their observed behaviors. To this end, we assume that higher memory players can directly imitate and adopt lower memory players’ strategies. In contrast, when lower memory players imitate a higher memory player, they adopt the low-memory strategy that is most compatible with the higher memory player’s behavior. For example, an unconditional player will imitate a memory-1 strategy’s average cooperation probability. Similarly, a reactive player will infer effective values of his strategy components p and q, which can also be calculated from the invariant distribution (see “reactive marginalization” in Park et al., “Cooperation in alternating interactions with memory constraints”, Nature Communications, 2022).

We find that this variation leads to a weakening of the memory dilemma. The payoffs of two players of different memory capacity tend to inch closer together as the imitation probability alpha increases. For our chosen value of alpha=0.05, this often leads to bistability of higher and lower memory in the evolutionary process for strong selection (instead of lower memory being stable). For weak selection we often find that reactive strategies become stable (instead of unconditional strategies).

***Changes:** We have amended the manuscript to include the above extension. We again visualize results in **Figs. S8 (e)** and **S9 (f)** for β=100. Moreover, we present the data for β=10 and β=100 in Tables **S14** and **S20**. We discuss this extension in the new subsection of the main text.*

Minor points:

1. How many times of simulation were performed for the tournaments? The authors should probably specify this in the Methods part if they have not.

***Reply and Changes**: Our tournament simulations were performed for 10^9 timesteps. We had specified this in the Methods section, but there it may be easily overlooked. Instead, we now also mention this value in the captions of the tables documenting the tournament results. Thank you for making us aware of this!*

2. The authors could expand their results or discussions a bit on strategies that require higher memory capacity. Although the strategy space grows dramatically with the memory capacity, using heuristics may help reduce the dimension of the strategy space. For example, people may not be able to remember exactly what a certain player chose in the last ten moves, but may probably have a rough sense of how often did he/she cooperate. What if agents with higher memory capacity use heuristic strategies (e.g., if the opponent often cooperate, I also cooperate)?

***Reply**: This is indeed an interesting suggestion. There is in fact some previous work that considers strategies that only count how often players cooperated, but not at which time points (cf. Hilbe et al., "Memory-n strategies of direct reciprocity", PNAS 2017). This research, however also suggests that such simplified strategies are less powerful. For example, they cannot encode behaviors that implicitly depend on the timing of cooperation. Yet, in some instances it may be important to know whether the two players did in fact cooperate (or defect) in the very same round. Thus, this research suggests that simplified strategies may be less effective in sustaining cooperation.*

***Changes:** We now discuss this issue in our Discussion section, thanks for bringing it up!*

3. It would be great if the authors could suggest some mechanisms that can potentially resolve the memory dilemma.

***Reply and Changes**: As our new findings on same-complexity sampling with averaging show (cf. **Fig S8, S9** as well as Tables **S13** and **S19**), decoupling the size of a strategy space from the players' memory capacity can let the memory dilemma vanish: constructing reactive strategies by sampling four values and averaging each two of them, and constructing unconditional strategies by averaging all four values, lets memory-1 win the competition. This happens because in this modification, lower memory strategies do not have the ability to find extreme behavior more quickly than memory-1. As mentioned above, we now additionally discuss this finding in the newly added subsection of our main text.*

4. Researchers in other fields including psychology and economics have also investigated the relationship between cognitive/memory capacity and the results of social interactions (e.g., Duffy & Smith, 2014). Discussions on the relationship between these studies and the current one in the introduction or discussion part may attract a wider audience for this paper.

Reference:

Duffy, S., & Smith, J. (2014). Cognitive load in the multi-player prisoner's dilemma game: Are there brains in games? Journal of Behavioral and Experimental Economics, 51, 47–56. http://doi.org/10.1016/j.socec.2014.01.006

***Reply and Changes**: We thank the reviewer for making us aware of this paper. It indeed makes a very interesting connection between cognitive load, memory/cognitive skills, and strategic game play. We have now incorporated a brief discussion of this work in the introduction of our manuscript.*

Reviewer #2:

Contribution:
This paper studies direct reciprocity through repeated donation games and has interesting findings on the behavioral strategies. It explores the role of memory capacity on cooperation. It touches base on an important assumption in the field of repeated game theory that history is perfectly memorized. In the main result, they identify three types of strategies spaces that differ in memory demands and found that smaller strategy spaces dominate larger ones. Overall I found the topic interesting but the paper itself needs to be a lot more clear in order to fit the interest to a broader audience. It needs to be explained in a much clearer way in order for it to be published in any journal. I feel it will be hopeless for someone to understand this manuscript if they don't read other articles on this set of notation. It is not that the math is complicated but just a matter of explanation. The authors must be aware that this journal is biological communication, most of the readers will have zero knowledge of game theory terminologies. I am afraid I cannot accept the paper unless the manuscript can be improved.

***Reply**: We thank the reviewer for their constructive criticism. We have now carefully revised our manuscript in order to improve clarity and readability for a broader audience.*

My specific suggestions are as follows:

The introduction part in my opinion is a bit too narrowed that it only touch base on this small circle of literature within memory-based games. As a person who doesn't belong to this literature, I don't get it immediately why we need to study this domain. Though personally, I think it's important. I just didn't get it from the writing. The author needs to say why this toy model they are simulating is important and how it maps to life-like scenarios. This is important as we can't just simulate something fun and make ourselves enthusiastic.

***Reply and Changes**: We agree that a more thorough motivation of our study would be indeed useful for out-of-field readers. Hence, we have now amended our Introduction to better reflect the relevance of this manuscript in a broader context. We have added applications of the Prisoner's Dilemma, and have briefly discussed experimental literature on the connection between working memory capacity and success in a repeated game.*

The basic structure of the game needs to be better visualized. It can be easily done through a table. I found figure 1 is even less helpful than the word (I saw they have a clear matrix in the dynamic part).

***Reply and Changes**: Thank you for making us aware of our insufficient explanation of the basic game. We now include the payoff matrix of the donation game in the results section to make this transparent and clear up any confusion.*

Starting from line 100, I found the notation very confusing and when a notation comes out for the first time, the authors are not explaining them clearly. One example can be, in line 103,

“we refer to ... set of strategies...”, here you need to explain the dimension of a strategy in mathematical terms along with its dimension. You can’t let readers infer according to your game structures. Where is the notation of history at this point if we are talking about strategies in a repeated game? Overall, I think the math doesn’t talk to intuition.

***Reply and Changes**: We appreciate the feedback. When revising our manuscript, we have been careful to more clearly explain strategy spaces, and how the game history and strategies are connected. We note that such a more formal explanation was previously included in the Methods section. We have now included the formal definitions of the three strategy spaces in the main text as well, for easier readability.*

The authors need to specify what limitation the PD game causes as it is a very specific type of cooperation that the cooperative strategy is not Nash equilibrium. If possible, I think authors can at least discuss what they think will happen in other domains of repeated games, not only PD.

***Reply**: This is a very good point. In our original version, we indeed entirely focused on the prisoner’s dilemma (or the donation game as a special case). We were particularly interested in this game because it is often regarded as the main paradigm to study reciprocity (see for example Hilbe et al., “Partners or rivals in direct reciprocity”, Nature Human Behaviour, 2018, or Sigmund, “The calculus of selfishness”, Princeton University Press, 2010).*

*However, our analysis is of course not limited to a single game matrix or structure. Rather, we agree that it is indeed of interest to explore the competition of different strategy/memory spaces in other repeated games beyond the PD. To this end, we now consider payoff matrices of the form {{1, u},{v,0}}. We vary u between [-1,1] and v between [0,2] (using a finite grid of possible values). Overall, we study 441 different game matrices. Matrices with u in [-1,0] and v in [1,2] are Prisoner’s Dilemmas. The other matrices can be classified into the fundamental social dilemmas Stag Hunt, Snowdrift, and Harmony games (**Fig.5a**). These three other structures can lead to different dynamics than the PD. We repeat our previous simulations for each of these 441 game matrices. Again, we explore which of the three memory spaces leads to the highest self-payoff. In addition, we again explore which memory space is the Nash equilibrium of the 3x3 payoff matrix when the three memory spaces compete. In this way, we find, for example, that other forms of repeated games can lead to “reverse” memory dilemmas. In such reverse dilemmas, lower memory gains the largest self-payoff, whereas higher memory is the Nash equilibrium.*

***Changes:** We have now amended the manuscript by adding two figures. These figures visualize the basic setup, and they provide an overview of our results for both weak and strong selection. The figure for strong selection (β=100) is included in the main text as **Fig.5**. In addition, **Fig. S10** shows the results for weak selection (β=10). We have also included a discussion of these new results in the Results section, and have revised the Discussion section accordingly.*

Reviewer #3:

This paper explores competition between different strategy spaces in the infinitely repeated donation game. The competition occurs between strategy spaces requiring different memory capacities, namely unconditional strategies (U), reactive strategies (R) and memory-1 strategies (M). Due to the asymmetry induced by competition between strategy spaces, the authors assume that behavioral update occurs via introspection dynamics throughout the course of the repeated game, rather than through e.g. imitation dynamics. They find that there is a tradeoff

between the quality of strategies available to a player with a given memory capacity and the ease with which high quality strategies can be found via introspection dynamics. They show that, depending on the strength of selection and the acuteness of the social dilemma encoded by the donation game, this tradeoff can lead to either U, R or M dominating. Most interestingly, they also explore replicator dynamics at level of the supergame and identify a "memory-dilemma" in which R evolves even though it does not maximize population-level payoff from cooperation.

In some ways the tradeoff at the heart of this model is a familiar one, which crops up frequently in evolutionary dynamics as a tension between robustness and evolvability. However that familiarity is not to the determinant of this work, and indeed the case the authors explore is especially fascinating due to the way it results in a social dilemma reproducing itself at different scales (i.e. the donation game formulation of the Prisoner's Dilemma becomes a Memory Dilemma in the supergame). I therefore believe that this paper will be of great interest to the wide readership of PLOS Computational Biology.

Reply: *Thank you for this favorable feedback!*

My only significant concern with the current manuscript is with the assumption that behavioral update occurs via introspection. This is a fine model to explore, but the central question here invites the reader to consider the implications for the evolution of memory, as the authors acknowledge in the discussion. It's not at all clear that introspection dynamics apply to e.g. competition between desert spiders (line 323). The reason the authors focus on introspection dynamics is (line 132-137) that a player with a lower memory capacity cannot imitate the strategy of a player with a higher memory capacity. However there is a very natural way that such imitation could happen: a U player could imitate an M player by "imitating" the average rate of cooperation of the M player. An R player could imitate an M player by imitating the average observed rate of cooperation of an M player in response to cooperation by an opponent, and so on. It seems likely that the reduced ability to accurately imitate induced by low memory capacity may undermine some of the results presented here, and I would be very interested for the authors to explore this.

Reply: *This is an excellent point, thank you for drawing our attention to this. Introspection dynamics is a basic model for how individuals with different memory capacity might revise their strategies. However, it is certainly true that the use of introspection dynamics is limited to select applications. Introspection dynamics assumes certain cognitive abilities, and thus it may not apply to all instances in which individuals learn to engage in reciprocity. In contrast, imitation or social learning is a very natural behavior. In evolutionary game theory, imitation processes are often taken as a standard model to describe the spread of strategies in a population. This makes it very worthwhile to explore in our model.*

We therefore now include a modification of our baseline model where we incorporate this suggestion. We have added a variation of our framework. In that variation, with a certain probability alpha, players imitation to revise their strategies. With (1-alpha), players use introspection dynamics. For this model to be sensible, one needs to assume that players are able to imitate their co-players' strategies from their observed behaviors. To this end, we thus assume that higher memory players can directly imitate and adopt lower memory players' strategies. In contrast, lower memory players imitate higher order strategies exactly in the way as suggested by the reviewer.

We find that this variation leads to a weakening of the memory dilemma over a wide range of cost values. The payoffs of two players of different memory capacity inch closer together as the imitation probability alpha increases. For our chosen value of alpha=0.05, this often leads

to bistability of higher and lower memory in the evolutionary process for strong selection (instead of lower memory being stable). For weak selection, we often find reactive strategies to be stable (instead of unconditional strategies).

Changes: *We have amended the manuscript accordingly. We visualize our results in **Figs. S8(e)** and **S9(f)** for β=100, and we present the data for β=10 and β=100 in **Tables S14** and **S20**. In addition, we discuss this extension in a newly added section of the main text.*
